# The Effects of *Poria cocos* Polysaccharides on Growth Performance, Immunity, and Cecal Microflora Composition of Weaned Piglets

**DOI:** 10.3390/ani14071121

**Published:** 2024-04-07

**Authors:** Jinzhou Zhang, Heming Wang, Shuaitao Meng, Chuankuan Zhang, Liping Guo, Zhiguo Miao

**Affiliations:** 1 College of Animal Science and Veterinary Medicine, Henan Institute of Science and Technology, No. 90, East Section of Hualan Avenue, Xinxiang 453003, China; zhangjz69@126.com (J.Z.); sqheming@163.com (H.W.); mengshuaitao2001@163.com (S.M.); chuankuan0526@163.com (C.Z.); 2 School of Food Science, Henan Institute of Science and Technology, No. 90, East Section of Hualan Avenue, Xinxiang 453003, China; lipingguo1982@126.com

**Keywords:** weaned piglet, growth performance, immunity, microflora, *Poria cocos* polysaccharide

## Abstract

**Simple Summary:**

Over recent decades, the rapid growth in the use of antibiotics in animal husbandry worldwide has increased awareness of their adverse effects, which pose high risks to public health. The fungus *Poria cocos* is commonly used as both a traditional Chinese medicine and has a broad range of effects, e.g., strengthening body resistance. Polysaccharides are the main bioactive components of *Poria cocos*. In this paper, we assessed the potential of *Poria cocos* polysaccharides (PCPs) as a feed additive for animal production and found that PCP treatment positively influenced the growth performance, immunity, and cecal microbiota composition in weaned piglets.

**Abstract:**

This paper aims to identify *Poria cocos* polysaccharides (PCPs) as a potential feed additive used for swine production; thus, we explored the effects of different dietary inclusion levels of PCP on growth performance, immunity, and cecal microflora composition in weaned piglets. For this, a total of 120 28-day-old Duroc × Landrace × Yorkshire weaned piglets (8.51 ± 0.19 kg; 28 ± 1 days of age) were randomly allocated to five groups that were fed a basal diet supplemented with 0, 0.025%, 0.05%, 0.1%, and 0.2% PCP, respectively, for 42 days. The results indicated that the average daily gain (ADG) and gain/feed ratio were higher in the PCP treatment groups than in the control group, with a linear effect. The serum concentrations of IgG, IgA, IL-2, IFN-γ, the number of CD4^+^ T cells, and the CD4^+^-to-CD8^+^ T-cell ratio (CD4^+^/CD8^+^) were increased, while the levels of IL-6 and TNF-α were decreased in the PCP supplementation groups compared with those in the control group. Furthermore, the cytokine mRNA expression levels exhibited a similar trend in the spleen. PCP supplementation also reduced the abundance of *Escherichia coli* and *Salmonella* and enhanced that of *Lactobacilli* and *Bifidobacteria* in the cecum. In summary, dietary PCP inclusion exerted positive effects on the growth performance, immunity, and cecal microbiota of piglets and showed potential for use as a feed additive for improving the health of weaned piglets, with 0.1% being the optimal dosage.

## 1. Introduction

Weaning is a critical time in the life of a pig. During this period, piglets face complex and stressful challenges, including maternal separation, feed conversion, environmental changes, and mixing with unfamiliar piglets [1]. These stress reactions lead to weakened immunity, indigestion, diarrhea, slowed growth, and even death in weaned piglets, resulting in heavy losses to swine farms [2]. Pig farmers have traditionally relied on the addition of antibiotics to alleviate weaning stress and prevent piglet diarrhea. Krishnasamy et al. [3] reported that 38.5 million kg of antibiotics were used in the Chinese pig and poultry industries in 2012. However, following market research on commonly used antibiotics in China, Zhang et al. [4] estimated that 48.4 million kg of antibiotics were consumed in the Chinese swine industry in 2013. Van Boeckelet al. [5] predicted that, by 2030, the worldwide use of antibiotics in food animals would reach 200.2 million kg, far exceeding that consumed by humans. However, accurately predicting antibiotic usage in the pig farming industry globally is a highly complex and unresolved task.

The rapid growth in the use of antibiotics in animal husbandry worldwide has increasingly highlighted their negative effects, such as environmental pollution and antibiotic resistance, which pose high risks to public health [6,7]. To address this concern, since 1 January 2006, a comprehensive legal ban on the use of antibiotics as growth promoters has been in force in the European Union, while on 1 July 2020, the Chinese government banned the use of antibiotics as additives in feed. These observations underscore the need to identify and develop alternatives to antibiotics in the livestock industry. One potential substitute for antibiotics is polysaccharides extracted from natural products. Studies have shown that natural polysaccharides have a wide range of properties, including immune regulatory, anti-inflammatory, and antioxidant activities; have good safety profiles; and are widely sourceable [8,9,10]. Chenet al. [11] reported that the addition of *Achyranthes bidentata* polysaccharides to the feed of weaned piglets enhanced the yield of complement (C3, C4), cytokines (interleukin-2 [IL-2], interferon-γ [IFN-γ]), and immunoglobulins (IgG, IgA, IgM) in the animals, thereby facilitating cellular and humoral immunity and affording significant protection against infection through nonspecific defense mechanisms. IFN-γ, tumor necrosis factor α (TNF-α), and IL-2 produced by Type 1 T helper (Th1) cells are in charge of the cell-mediated immunity against intracellular pathogens, while IL-4 and IL-10 produced by Th2 cells are in charge of the promotion of antibody responses [12]. Wang et al. [8] found that *Angelica* and *Radix isatidis* polysaccharides could mitigate cyclophosphamide-induced immunosuppression in Sprague–Dawley rats, as well as improve their weight restoration and spleen indexes, suggesting that the two polysaccharides have the potential for use as raw material resources for novel veterinary drugs. In a different study, the authors found that the polysaccharide extracted from the mushroom *Caripia montagnei* exerted anti-inflammatory effects on rats with carrageenan-induced pleuritis. Moreover, Zhang et al. [10] confirmed that the dietary supplementation of Gan cao polysaccharides improved the average daily weight gain and the profile of the cecal microflora in broilers.

Polysaccharides are the major bioactive components of the fungus *Poria cocos* (Schw.), commonly used as both a traditional Chinese medicine and food. β-Glucan, the major *Poria cocos* polysaccharide (PCP), possesses a β-(1→3)-linked glucose backbone and β-(1→6)-linked glucose side chains [13]. PCP has a wide range of pharmacological effects, including as an immune enhancer, and has wide prospects for application as a prebiotic in the animal industry [14,15]. Xu et al. [16] found that PCP played an immune-regulatory role in mice, alleviating antibiotic-related diarrhea by modulating the homeostasis of intestinal microorganisms and the gut mucosal barrier. Dong et al. [17] also reported that PCP enhanced cellular Th1 immune responses. However, no study to date has evaluated the effect of *Poria cocos* polysaccharides on the growth performance, immunity, and cecal microbiota composition in pigs.

The above results suggested that the inclusion of PCP in the diet may exert positive effects on growth and immunity in weaned piglets. Accordingly, in this study, we investigated the influence of different PCP dietary inclusion levels on growth performance, immunity, and cecal microflora composition in weaned piglets, aiming to identify a potential feed additive for use in swine production.

## 2. Materials and Methods

### 2.1. Ethical Statement

All animal experiments were approved by the Animal Protection and Utilization Committee of the Henan Academy of Science and Technology (approval number 2023HIST018, Xinxiang, China).

### 2.2. Poria cocos Polysaccharide Preparation

*Poria cocos* polysaccharide was purchased from Hubei Huisheng Biotechnology Co., Ltd. (Wuhan, China) as a grayish-white powder with a drying weight loss of less than 5% and a polysaccharide content ≥90%.

### 2.3. Animals, Trial Design, and Management

A total of 120, healthy, 28-day-old Duroc×Landrace×Yorkshire (DLY) weaned piglets were provided by Chunfa Farm in Shenqiu County, Henan, China. The basal diet for the experiment was formulated to meet the NRC (2012) nutritional standard for weaned piglets. Its composition and nutritional level are displayed in Table 1. The piglets were randomly allocated to five groups, namely, a control group, in which the piglets were fed the basal diet, and four PCP groups, in which the animals were fed the basal diet supplemented with 0.025%, 0.05%, 0.1%, and 0.2% PCP. This study used a feed mixer to mix PCP and feed. Firstly, the required amount of PCP for each group was mixed evenly with the 10% of the feed and then mixed evenly with the remaining feed. Each group had three replicates, with eight piglets per replicate (sex-balanced). The animals were raised in a pigsty in an artificially controlled environment, one replicate (eight piglets) per pigpen, and allowed free access to feed and water. The feeding management was performed according to the piggery’s procedures. The experiment lasted for 49 days, including a 7-day pretrial period. The first 7 days were the pretrial period. During the pretrial period, the pigs adapted to new pens, new populations, and reduced stress and only a basal diet was fed. The last 42 days were the trial period; five groups were fed the basal diet, or the basal diet supplemented with 0.025%, 0.05%, 0.1%, and 0.2% PCP, respectively.At the beginning and end of the experiment, the piglets were fasted for 12 h (with free access to drinking water) and weighed to determine the initial body weight (IBW) and final body weight (FBW), respectively. Daily feed consumption and residual feed were recorded during the experiment, and the values were used to calculate the average daily gain (ADG), average daily feed intake (ADFI), and gain/feed ratio.

### 2.4. Sample Collection

#### 2.4.1. Serum Collection

On the morning of trial day 43, two piglets (one male and one female, close to the replicate average weight) were chosen from each replicate for blood collection. Blood (10 mL) was collected from the anterior vena cava either into sterile tubes (5 mL, for immunoglobulin and cytokine concentration analyses) or vacuum tubes containing heparin (5 mL, for lymphocyte isolation). The non-anticoagulated blood samples were placed at room temperature for 1h and centrifuged at 1500× *g* for 15 min. The serum was carefully collected and stored at −20 °C for serum index analysis.

#### 2.4.2. Lymphocyte Isolation

Lymphocytes were isolated from heparinized blood samples by density gradient centrifugation following the manufacturer’s instructions (Solarbio, Beijing, China). After centrifugation at 600× *g* for 30 min at room temperature, the lymphocytes were aspirated into new centrifuge tubes, washed three times with PBS with centrifugation at 250× *g* for 10 min, and then resuspended in RPMI-1640 containing 5% fetal bovine serum (HyClone, UT, USA) to a density of 1 × 10^7^ cells/mL for use in flow cytometry.

#### 2.4.3. Tissue Sample Collection

After blood collection, the piglets were euthanized using electrical stunning methods. The spleen was subsequently separated from visceral organs, immediately placed in liquid nitrogen, and then placed at −80 °C in 1.5 mL sterile tubes for later analysis.

#### 2.4.4. Intestinal Sample Collection

After euthanasia, the cecal contents were collected from the same location in each animal into sterile tubes, placed in an ice box, and transported back to laboratory for microbial analysis.

### 2.5. Determination of Immunoglobulin and Cytokine Contents and the CD4^+^/CD8^+^ T Cell Ratio

Immunoglobulin (Ig) and cytokine concentrations in serum were determined using enzyme-linked immunosorbent assays (ELISAs) following the kits’ instructions of therespective kits (Nanjing Jiancheng Bioengineering Institute, Nanjing, China). The immune indicators tested included IgA, IgG, IgM, IL-1β, IL-2, IL-6, IL-10, IFN-γ, and tumor necrosis factor-alpha (TNF-α).

The ratio of CD4^+^ to CD8^+^ T lymphocytes was assessed by flow cytometry according to Lestari et al. [18]. Briefly, 1 × 10^6^ cells per sample were added to a 1.5 mL centrifuge tube and incubated with 2 μL of anti-CD4α-FITC, anti-CD8α-R-PE, and anti-CD3ε-SPRD antibodies (SouthernBiotech, Birmingham, AL, USA) at 4 °C in the dark for 30 min. Subsequently, the suspensions were centrifuged, the supernatant was discarded, and the cells were washed three times with PBS, resuspended in 300 μL of PBS, and subjected to flow cytometric analysis using a FACSCalibur (Becton, Dickinson and Company, New Jersey, NJ, USA).

### 2.6. Measurement of Cytokine mRNA Levels in the Spleen

Total RNA was extracted from 50 mg of spleen tissue from each sample with TRIzol Reagent (Invitrogen, Carlsbad, CA, USA) following the instructions of the manual and then reverse-transcribed using an M-MuLV First Strand cDNA Synthesis Kit (Sangon Biotech, Shanghai, China). The expression levels of cytokines in spleen tissue were assessed by quantitative real-time PCR (qPCR) and calculated using the 2^−ΔΔCt^ method [19]; β-actin served as the housekeeping gene. The sequences of the primer pairs used for qPCR are shown in Table 2.

### 2.7. Analysis of the Cecal Microflora

Analysis of the cecal microflora was performed as described by Zhang et al. [10]. Cecal contents (0.5 g) were placed in a sterile test tube to which 5 mL of sterile normal saline was added. After shaking for 10 min, the suspension was diluted 10-fold and then further serially diluted with sterile normal saline from 1 × 10^2^ to 1 × 10^8^. Subsequently, 0.1 mL of the 10^5^, 10^6^, 10^7^, and 10^8^ dilutions were plated on Petri dishes (three replicates per dilution). *Lactobacilli*, *Bifidobacteria*, *Escherichia coli*, and *Salmonella* were anaerobically cultured at 37 °C for 48 h on Man–Rogosa–Sharpe agar, BBL, eosin methylene blue agar, and MacConkey agar, respectively. Finally, bacterial units were recorded and expressed as the number of colony-forming units (CFUs) per 1g of cecal sample.

### 2.8. Statistical Analysis

The data were analyzed using SPSS 25.0 software. The significance of differences among the five groups was assessed using one-way ANOVA followed by Duncan’s post hoc test. The data are presented as means ± standard error of the mean (SEM), with *p* < 0.05 indicating significant differences among the groups.

## 3. Results

### 3.1. The Effect of PCP on Growth Performance

The effect of dietary PCP supplementation on weight gain in weaned piglets is presented in Table 3. The results showed that adding different levels of PCP to the diet improved the ADG. Meanwhile, the different doses of PCP exerted differential effects on the weight of the piglets. As shown in Table 3, the ADFI was not affected (*p* = 0.683) by dietary PCP administration; in contrast, both the ADG (*p* < 0.05) and gain/feed ratio (*p* < 0.05) were significantly improved in piglets receiving 0.05%, 0.1%, and 0.2% PCP in the diet, except for 0.025% PCP; furthermore, they were linearly correlated with the amount of PCP supplementation (*p* = 0.021; *p* = 0.035). Overall, the addition of 0.1% PCP exerted the best effect on growth performance in the weaned piglets.

### 3.2. The Effect of PCP on Serum Immunoglobulin and Cytokine Contents

The influence of dietary PCP supplementation on serum Ig and cytokine contents in weaned piglets is shown in Table 4. We found that the dietary supplementation of 0.05%, 0.1%, and 0.2% PCP markedly increased serum IgG and IgA concentrations (*p* < 0.05); the serum IgG concentration linearly increased with the dietary supplementation of PCP (*p* = 0.009) but did not influence IgM levels (*p* = 0.423). Meanwhile, PCP supplementation did not affect the serum levels of IL-1β (*p* = 0.541) or IL-10 (*p* = 0.134). However, compared with the control condition, dietary PCP increased the serum concentrations of IL-2 (*p* < 0.05; 0.025%, 0.05%, 0.1%, and 0.2% PCP) and IFN-γ (*p* < 0.05; 0.05%, 0.1%, and 0.2% PCP) and decreased those of IL-6 (*p* < 0.05; 0.05%, 0.1%, and 0.2% PCP) and TNF-α (*p* < 0.05; 0.1% and 0.2% PCP); the serum IL-2 (*p* = 0.011), IFN-γ (*p* = 0.047), and TNF-α (*p* = 0.030) levels were linearly correlated with the amount of PCP supplementation. These data demonstrated that the dietary inclusion of 0.1% and 0.2% PCP (*p* < 0.05) improved serum immune indexes in the weaned piglets.

### 3.3. The Effect of PCP on the CD4^+^/CD8^+^ T Cell Ratio

As shown in Table 5, there were no differences in the number of CD3^+^ (*p* = 0.268) or CD8^+^ (*p* = 0.163) lymphocytes among the groups. The number of CD4^+^ lymphocytes and the CD4^+^/CD8^+^ ratio were higher both in the 0.1% and 0.2% PCP treatment groups than in the control group (*p* < 0.05), and they linearly increased with the dietary supplementation of PCP (*p* = 0.046; *p* = 0.025). The dietary supplementation of 0.1% and 0.2% PCP (*p* < 0.05) exerted the best effect on the CD4^+^/CD8^+^ ratio in weaned piglets.

### 3.4. The Effect of PCP on Cytokine mRNA Levels in the Spleen

**Figure 1:**
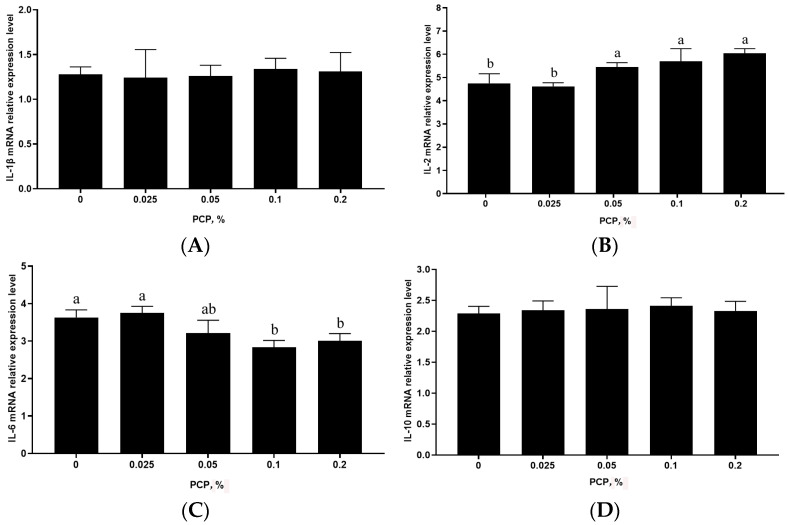

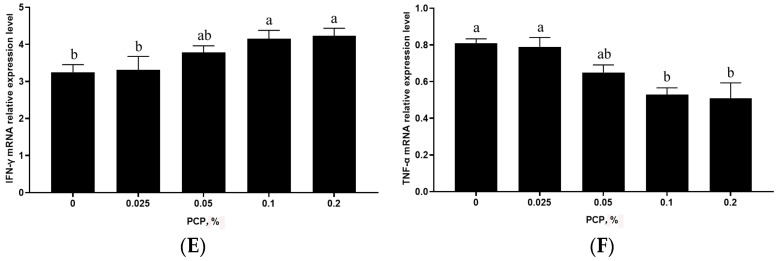
The effect of PCP supplementation on cytokine mRNA levels in the spleen of weaned piglets. (**A**) *IL-1β*; (**B**) *IL-2*; (**C**) *IL-6*; (**D**) *IL-10*; (**E**) *IFN-γ*; (**F**) *TNF-α*. *β-actin* served as the housekeeping gene. In the figure, different lowercase letters above the columns denote significant differences (*p* < 0.05).

The mRNA levels of cytokines in the spleen of weaned piglets, as detected by qPCR, are displayed in Figure 1. The mRNA levels of *IL-1β* (*p* = 0.214) and *IL-10* (*p* = 0.165) in the spleen were not affected by dietary PCP administration. However, compared with the control group, the mRNA levels of *IL-6* and *TNF-α* were down regulated in the 0.1% and 0.2% PCP treatment groups (*p* < 0.05), and they were linearly correlated with the amount of PCP supplementation (*IL-6*: *p* = 0.021, *p* = 0.035; *TNF-α*: *p* = 0.010, *p* = 0.016), while those of *IL-2* in the 0.25%, 0.1%, and 0.2% PCP treatment groups and of *IFN-γ* in the 0.1% and 0.2% PCP treatment groups were upregulated (*p* < 0.05), and they linearly increased with the dietary supplementation of PCP (*IL-2: p* = 0.032, *p* = 0.022, *p* = 0.045; *IFN-γ: p* = 0.006, *p* = 0.001). Overall, the 0.1% and 0.2% PCP concentrations (*p* < 0.05) exerted the greatest influence on the mRNA expression levels of cytokines in the spleen of weaned piglets (Figure 1).

### 3.5. The Effect of PCP on the Cecal Microflora

The abundance of *Escherichia coli*, *Salmonella*, *Lactobacilli*, and *Bifidobacteria* in the cecum of piglets in the PCP treatment groups displayed a changing trend relative to that in the control group (Table 6). The abundance of *Escherichia coli* and *Salmonella* was significantly reduced, and they linearly decreased with the dietary supplementation of PCP (*p* = 0.020; *p* = 0.012), whereas that of *Lactobacilli* and *Bifidobacteria* was significantly increased in the 0.05%, 0.1%, and 0.2% PCP treatment groups (*p* < 0.05). In summary, the addition of PCP to the diet positively influenced the cecal microbial profile, with the 0.1% PCP concentration producing the best effect.

## 4. Discussion

In this work, we explored the potential for the use of PCP as a feed additive in the pig-rearing industry and presented valuable data regarding the effects of PCP on the growth, serum immune indexes, expression of immune-related genes, and cecal microbiota composition of weaned piglets. Our results indicated that the addition of appropriate concentrations of PCP to the diet can increase the growth performance of weaned piglets with a linear effect. Growth performance is the core indicator for evaluating the success of pig farm management, as increased growth results in higher yields and greater economic benefits. Hence, how to best improve growth efficiency in pigs has become a hot topic of discussion for piggeries. However, there is a lack of information regarding the impact of PCP on porcine growth performance in the literature; recent studies have shown that dietary PCP supplementation exerts a positive effect on the growth performance in Dabry’s sturgeons and spotted sea bass, with suggested optimal dosages of 0.2 and 1.2 g/kg, respectively [20,21]. Furthermore, PCP administration was reported to mitigate the 5-fluorouracil treatment-induced body weight reduction in *Apc^Min/+^* mice [14]. Combined, these data confirmed that PCP has a promotive effect on animal growth, which is expected to improve the economic benefits of pig farms, and more studies focusing on the underlying molecular pharmacological mechanism are warranted.

Recent studies have shown that the bioactive components of *Poria cocos* are polysaccharides, triterpenoids, fatty acids, sterols, etc.; PCP accounts for 84% of bioactive components in dried *Poria cocos* and can act as an immune enhancer, improving both humoral and cellular immunity [13,22,23,24]. Serum IgG, IgA, and IgM play key roles in immune function, and their concentrations can reflect the health status of pigs. Jiang et al. [25] found that feeding PCP to normal mice boosted the serum contents of IgA, IgM, and IgG, and this effect was dose-dependent. Liu et al. [26] found that the addition of PCP to the diet significantly elevated serum IgG and IgM levels in immunosuppressed mice and contributed to the restoration of the suppressed humoral immune function by modulating the TLR4/NF-κB signaling pathway. In this study, we found that dietary PCP addition significantly increased the serum concentrations of IgG and IgA in weaned piglets, which is in line with the abovementioned findings. However, PCP supplementation did not influence the levels of IgM, an observation that requires further exploration.

Cytokines are low-molecular-weight soluble proteins produced by a wide variety of cell types andserving as important mediators of immune responses [27]. A recent study showed that *Cordyceps sinensis* polysaccharides promoted cytokine secretion (IFN-γ, TNF-α, IL-2, IL-4, IL-6, IL-10, IL-12, IL-13, IL-17, and IL-21) in cyclophosphamide-treated mice and had a protective effect on intestinal mucosal immune suppression [28]. Liu et al. [29] found that *Sanghuangporus vaninii* polysaccharides significantly reduced the levels of proinflammatory IL-1β, IL-6, and TNF-α and elevated those of anti-inflammatory IL-10 in lipopolysaccharide-treated RAW 264.7 cells. Alagbaoso and Mizuno [30] reported that *Lentinula edodes* polysaccharides greatly alleviated weight loss in mice with dextran sulfate sodium-induced colitis and inhibited the expression of proinflammatory cytokines (TNF-α, IL-1β, IL-6, and IFN-γ), suggesting that the polysaccharide may be effective for treating inflammatory bowel disease. In this study, we found that dietary PCP administration elevated the serum levels of IL-2 and IFN-γ, while reducing those of IL-6 and TNF-α in weaned piglets; no effect of PCP was detected on the serum contents of IL-1β and IL-10. Furthermore, the mRNA levels of these cytokines exhibited a similar trend in the spleen. However, our results relating to the effects of PCP treatment on cytokine levels in weaned piglets are inconsistent with those of previous works [28,31], which may be associated with the pleiotropy and complexity of cytokines.

CD4^+^ and CD8^+^T cells participate in the recognition of foreign antigens and the clearing of cells that have been invaded by viruses or bacteria [32]. The CD4^+^/CD8^+^ ratio is an important indicator of the level of cellular immunity, and an increase in this ratio in blood reflects an improvement in animal immunity [33]. Our results showed that the dietary inclusion of 0.1% and 0.2% PCP exerted the greatest effects on improving the CD4^+^ T cell count and the CD4^+^/CD8^+^ ratio in weaned piglets relative to that in the control group, implying that PCP is effective at elevating cellular immunity in weaned piglets. Shan et al. [34] found that *Schisandra chinensis* polysaccharides moderated the secretion of numerous inflammatory factors and increased the CD4^+^/CD8^+^ T cell ratio in mice with concanavalin A-induced immune-mediated liver injury. A different study reported that *Atractylodes macrocephala* Koidz. polysaccharides elevated the CD4^+^/CD8^+^ lymphocyte ratio in chickens and had a notable regulatory effect on cellar immunity [35]. Our results are consistent with these findings. 

In our study, we assessed the abundance offour classic bacteria—*Escherichia coli*, *Salmonella*, *Lactobacilli*, and *Bifidobacteria*—in the cecum of weaned piglets. Some studies have shown that weaning has a significant impact on the gut microbiota of piglets, mainly manifesting as an increase in the abundance of *Escherichia coli* and a decrease in that of *Lactobacilli*, leading to piglet intestinal dysfunction and diarrhea [36,37,38]. Potential pathogens such as *Escherichia coli* and *Salmonella* produce endotoxins and other substances in the animal intestine under stress, and these factors damage the intestinal barrier and disrupt intestinal homeostasis, thereby causing diarrhea [16]. In contrast, beneficial bacteria such as *Lactobacilli* and *Bifidobacteria* can produce organic acids through the fermentation of substrates such as fructose, starch, and glycogen, leading to a decrease in pH in the intestinal contents and thereby suppressing the proliferation of acid-sensitive pathogenic microorganisms [39]. Moreover, *Lactobacilli* and *Bifidobacteria* can adhere to the intestinal epithelium and suppress the growth of pathogenic bacteria by competing with them for adhesion sites [40]. Our findings showed that PCP treatment reduced the abundance of *Escherichia coli* and *Salmonella* and enhanced that of *Lactobacilli* and *Bifidobacteria*, which agreed with previous studies [39,40]. These results indicated that PCP can ameliorate the composition of the microbial population in the cecal contents of weaned piglets, thus enhancing intestinal health.

## 5. Conclusions

In summary, the dietary supplementation of PCP positively influenced growth performance, immunity, and cecal microbiota composition in weaned piglets. *Poria cocos* polysaccharides can be used as a feed additive to improve the health of weaned piglets, with 0.1% being the optimal inclusion level.

## Figures and Tables

**Table 1:** The ingredients and nutrient levels of the experimental diets.

Ingredients	Content, %	Nutrient Levels ^2^	Content, %
Corn	59.4	Digestible energy (MJ/kg)	13.62
Soybean meal	19.7	Crude protein (%)	19.58
Expanded soybean	8.0	Crude fat (%)	3.90
Whey	3.6	Calcium (%)	0.91
Soya oil	2.0	Available phosphorus (%)	0.62
Fish meal	3.0	Lysine (%)	1.48
NaCl	0.3		
Premix ^1^	4.0		

^1^ The premix provided the following per kg of diet: vitamin A, 9750 IU; vitamin D_3_, 3000 IU; vitamin B_1_, 3 mg; vitamin B_2_, 3.7 mg; vitamin B_6_, 2 mg; vitamin E, 22.5 mg; vitamin K_3_, 3 mg; folic acid, 1.5 mg; nicotinic acid, 30 mg; pantothenic acid, 15 mg; Cu (as copper sulfate), 5 mg; Fe (as ferrous sulfate), 80 mg; I (as potassiumiodide), 0.14 mg; Mn (as manganese sulfate), 20.5 mg; Se (as sodium selenite), 0.15 mg; and Zn (as zinc sulfate), 80 mg. ^2^ Nutrient level was calculated according to the Tables of Feed Composition and Nutritive Values in China (2015, twenty-sixthedition).

**Table 2:** Detailed information for the primersused for qPCR analysis of cytokine genes.

Gene	ID	Sequence (5′–3′)	Annealing, °C	Extension, S	Product Length, bp
IL-1β	NM_214055	GCTGGATGCTCCCATTTCTCA GAAAGCCCAATTCAGGGACCC	60	40	217
IL-2	JN851821	AACTGTAAATCCAGCAGCAAT ACAATGGGTAAGATGCAGCTC	60	40	131
IL-6	AF518322	TTTGCCGAGGATGTACTTAA ATGAACTCCCTCTCCACAAGC	60	40	190
IL-10	HQ236499	ACGCCCATCTGGTCCTTCGTT ATGCCCAGCTCAGCACTGCTC	60	40	176
TNF-α	JF831365	CGGGCTTATCTGAGGTTTGAG GACACCATGAGCACTGAGAGC	60	40	268
IFN-γ	NM_213948	AAAAGAGGTCCACCATTAGG CAGAAGCTAACTCTCTCCGAA	60	40	179
β-actin	U07786	CGTGGTGGTGAAGCTGTAGCC ATGTTTGAGACCTTCAACACGC	60	40	243

Abbreviations: IL-1β, interleukin-1β; IL-2, interleukin-2; IL-6, interleukin-6; IL-10, interleukin-10; IFN-γ, interferon γ; TNF-α, tumor necrosis factor α.

**Table 3:** The effect of PCP supplementation on the growth performance of weaned piglets.

	PCP Level, %						*p*-Value		
Item	0	0.025	0.05	0.1	0.2	SEM	Groups	Linear	Quadratic
IBW, kg	8.51	8.58	8.46	8.48	8.52	0.126	0.983	0.842	0.968
FBW, kg	24.34 ^b^	24.81 ^b^	25.72 ^ab^	26.31 ^a^	26.13 ^a^	0.641	0.031	0.066	0.311
ADG, g/d	376.90 ^c^	386.43 ^c^	410.95 ^b^	424.52 ^a^	419.29 ^a^	13.332	0.001	0.021	0.239
ADFI, g/d	723.07	732.84	722.44	731.19	734.52	35.403	0.683	0.304	0.601
Gain/feed ratio	0.52 ^b^	0.53 ^b^	0.57 ^a^	0.58 ^a^	0.57^a^	0.002	0.001	0.031	0.467

Abbreviations: ADFI, average daily feed intake; ADG, average daily gain; FBW, final body weight; IBW, initial body weight; PCP, *Poria cocos* polysaccharides; SEM, standard error of the means (n = 24). In the same row, values with different lowercase letters differ significantly from each other (*p* < 0.05).

**Table 4:** The effect of PCP supplementation on the serum immune profile of weaned piglets.

	PCP Level, %						*p*-Value		
Item	0	0.025	0.05	0.1	0.2	SEM	Groups	Linear	Quadratic
IgG (g/L)	3.45 ^c^	4.66 ^b^	5.16 ^a^	5.48 ^a^	5.61 ^a^	0.130	0.001	0.009	0.878
IgA (g/L)	0.20 ^b^	0.27 ^ab^	0.33 ^a^	0.34 ^a^	0.33 ^a^	0.051	0.028	0.298	0.456
IgM (g/L)	0.67	0.64	0.67	0.70	0.65	0.011	0.423	0.654	0.751
IL-1β (ng/L)	2.33	2.26	2.37	2.41	2.34	0.156	0.541	0.342	0.611
IL-2 (ng/L)	36.51 ^c^	40.46 ^b^	42.64 ^a^	44.60 ^a^	44.63 ^a^	1.317	0.005	0.011	0.722
IL-6 (ng/L)	16.45 ^a^	15.61 ^ab^	14.29 ^b^	14.33 ^b^	14.38 ^b^	0.803	0.030	0.053	0.675
IL-10 (ng/L)	11.23	11.47	12.01	11.56	11.29	0.396	0.134	0.546	0.203
IFN-γ (ng/L)	24.34 ^c^	24.52 ^c^	26.35 ^b^	28.01 ^a^	28.16 ^a^	1.014	0.015	0.047	0.410
TNF-α (ng/L)	0.63 ^a^	0.51 ^ab^	0.47 ^ab^	0.43 ^b^	0.41 ^b^	0.023	0.016	0.030	0.313

Abbreviations: Ig A, immunoglobulin A; Ig G, immunoglobulin G; Ig M, immunoglobulin M; IL-1β, interleukin-1β; IL-2, interleukin-2; IL-6, interleukin-6; IL-10, interleukin-10; IFN-γ, interferon γ; TNF-α, tumor necrosis factor α; PCP, *Poria cocos* polysaccharides; SEM, standard error of the means (n = 6). In the same row, values with different lowercase letters differ significantly from each other (*p* < 0.05).

**Table 5:** The effect of PCP supplementation on the percentage of lymphocyte subsets and the ratio of CD4^+^/CD8^+^ T cells in weaned piglets.

	PCP Level, %						*p*-Value		
Item	0	0.025	0.05	0.1	0.2	SEM	Groups	Linear	Quadratic
CD3^+^	65.44	67.30	65.41	66.15	66.07	4.243	0.268	0.381	0.524
CD4^+^	41.19 ^b^	41.72 ^b^	43.56 ^ab^	45.20 ^a^	45.34 ^a^	2.327	0.021	0.046	0.365
CD8^+^	33.45	33.51	34.05	34.23	34.11	2.134	0.163	0.113	0.201
CD4^+^/CD8^+^	1.23 ^b^	1.25 ^b^	1.28 ^ab^	1.32 ^a^	1.33 ^a^	0.057	0.036	0.025	0.337

Abbreviations: CD4^+^/CD8^+^, ratio of CD4^+^ to CD8^+^ of lymphocytes; PCP, *Poria cocos* polysaccharide; SEM, standard error of the means (n = 6). In the same row, values with different lowercase letters differ significantly from each other (*p* < 0.05).

**Table 6:** The effect of PCP supplementation on the cecal microflora of weaned piglets (log10 cfu/g).

	PCP Level, %						*p*-Value		
Item	0	0.025	0.05	0.1	0.2	SEM	Groups	Linear	Quadratic
*Lactobacilli*	6.58 ^b^	6.85 ^ab^	7.20 ^a^	7.41 ^a^	7.36 ^a^	0.151	0.014	0.109	0.351
*Bifidobacteria*	7.32 ^c^	7.39 ^c^	7.88 ^b^	8.34 ^a^	8.31 ^a^	0.175	0.021	0.074	0.233
*Escherichia coli*	5.24 ^a^	5.01 ^a^	4.62 ^b^	4.45 ^b^	4.38 ^b^	0.086	0.011	0.020	0.425
*Salmonella*	4.81 ^a^	4.56 ^b^	4.33 ^b^	3.50 ^c^	3.44 ^c^	0.062	0.003	0.012	0.316

Abbreviations: PCP, *Poria cocos* polysaccharide; SEM, standard error of the means (n = 6). In the same row, values with different lowercase letters differ significantly from each other (*p* < 0.05).

## Data Availability

The raw data supporting the conclusions of this article will be made available by the authors without undue reservation.
